# The role of a pre-load beverage on gastric volume and food intake: comparison between non-caloric carbonated and non-carbonated beverage

**DOI:** 10.1186/1475-2891-10-114

**Published:** 2011-10-14

**Authors:** Rosario Cuomo, Maria Flavia Savarese, Giovanni Sarnelli, Emanuele Nicolai, Adriana Aragri, Carla Cirillo, Letizia Vozzella, Francesco Paolo Zito, Viviana Verlezza, Eleonora Efficie, Maxime Buyckx

**Affiliations:** 1 Gastroenterology Unit, Department of Clinical and Experimental Medicine, University of Naples, Italy; 2 IRCCS- SDN Foundation, Naples, Italy; 3 The Coca Cola Company, Atlanta, USA

**Keywords:** Carbonated beverage, gastric volume, calorie intake, liquid meal, solid meal, ghrelin, cholecystokinin

## Abstract

**Background:**

There is conflicting data on the effects of carbon dioxide contained in beverages on stomach functions. We aimed to verify the effect of a pre-meal administration of a 300 ml non-caloric carbonated beverage (B+CO_2_) compared to water or a beverage without CO_2 _(B-CO_2_), during a solid (SM) and a liquid meal (LM) on: a) gastric volume, b) caloric intake, c) ghrelin and cholecystokinin (CCK) release in healthy subjects.

**Methods:**

After drinking the beverages (Water, B-CO_2_, B+CO_2_), ten healthy subjects (4 women, aged 22-30 years; BMI 23 ± 1) were asked to consume either an SM or an LM, at a constant rate (110 kcal/5 min). Total gastric volumes (TGV) were evaluated by Magnetic Resonance Imaging after drinking the beverage and at maximum satiety (MS). Total kcal intake at MS was evaluated. Ghrelin and CCK were measured by enzyme immunoassay until 120 min after the meal. Statistical calculations were carried out by paired T-test and analysis of variance (ANOVA). The data is expressed as mean ± SEM.

**Results:**

TGV after B+CO_2 _consumption was significantly higher than after B-CO_2 _or water (p < 0.05), but at MS, it was no different either during the SM or the LM. Total kcal intake did not differ at MS after any of the beverages tested, with either the SM (Water: 783 ± 77 kcals; B-CO_2_: 837 ± 66; B+CO_2_: 774 ± 66) or the LM (630 ± 111; 585 ± 88; 588 ± 95). Area under curve of ghrelin was significantly (p < 0.05) lower (13.8 ± 3.3 ng/ml/min) during SM following B-CO_2 _compared to B+CO_2 _and water (26.2 ± 4.5; 27.1 ± 5.1). No significant differences were found for ghrelin during LM, and for CCK during both SM and LM after all beverages.

**Conclusions:**

The increase in gastric volume following a 300 ml pre-meal carbonated beverage did not affect food intake whether a solid or liquid meal was given. The consistency of the meal and the carbonated beverage seemed to influence ghrelin release, but were unable, under our experimental conditions, to modify food intake in terms of quantity. Further studies are needed to verify if other food and beverage combinations are able to modify satiation.

## Introduction

Today, sweetened carbonated beverages are widely consumed and this has fuelled several conflicting opinions regarding their effect on satiation and food intake [1]. There is inconsistent data regarding the effects of carbon dioxide contained in beverages on the upper digestive tract [2,3]. The carbon dioxide contained in these beverages could increase gastric volume, consequently inducing a feeling of epigastric discomfort; therefore, carbonated drinks could determine early satiety. However, Zachwieja et al showed that adding carbonation to a drink does not significantly alter either the gastric function or the perception of gastrointestinal discomfort [4]. Pouderoux et al. also found no difference in gastric emptying or in the feeling of fullness between 300 ml of both carbonated and still water, drunk together with a 700 kcal meal, but showed an increased need to belch after consuming carbonated water [5]. Similar results emerged from a study carried out on healthy volunteers where 300 ml of sweetened beverages with or without added carbon dioxide did not influence the gastric emptying of a standard 480 kcal meal [6]. Contrasting results come from two other studies. In the first one, an 800 ml intake of either water or regular cola determined increased gastric distress and delayed gastric emptying [7]. The second study showed that a pre-load beverage of regular cola determined an initial increase in satiety without any reduction in energy intake after drinking the beverage [8]. Other studies showed that increasing the amount of carbonated beverage up to 400 ml seemed to limit energy intake during the meal or increase gastric distress [9,10]

Meal consistency also affects energy intake. Energy consumed in a liquid form has been repeatedly shown to result in a lesser reduction in hunger than the same energy load ingested in solid form [11]. Wadden et al. [12] noted a greater feeling of hunger in subjects given a 420-kcal high protein liquid diet, than in those who consumed a 400-kcal diet of lean fish, meat and fowl. No comparative data exist on the same subject regarding the effect of a carbonated beverage on the intake of a meal with different consistency (i.e., liquid or solid).

Moreover, various gastrointestinal hormones play key roles in determining satiety or hunger. Cholecystokinin (CCK) is an established satiety factor which binds to the CCK-1 and -2 receptors concentrated in the gut and the brain, respectively [13]. Conversely, ghrelin is an orexigenic peptide largely produced by the "X/A-like" cells of the oxyntic glands of the stomach and a ligand for growth hormone secretagogue receptors [14,15]. There is no consistent data regarding the effect of carbonated beverages on gastrointestinal hormone secretion following the intake of either a solid or a liquid meal.

One of the most widely used experimental methods to study the regulation of food intake is the preload-test meal paradigm [16]. Utilizing this method, we aimed to verify the effect of a pre-meal administration of a non-caloric carbonated beverage with respects to water and de-gassed non-caloric carbonated beverage on: a) gastric volume, b) caloric intake, c) gastrointestinal symptoms and eating perceptions, d) ghrelin and CCK release, in healthy subjects during standardized solid and liquid meals.

## Subjects and Methods

### Subjects

Ten healthy volunteers (4 women, 6 men; median age 22 years; range 19-24 years) without any gastrointestinal symptoms at the time of enrolment or previous gastrointestinal illness were recruited. Their mean body mass index (BMI) was 23 ± 1 (see Table 1). Exclusion criteria included: altered biochemical analysis; prior abdominal surgery; presence of gallbladder stones; positive symptoms at the dyspeptic or bowel symptom questionnaire; use of medications known to alter gastrointestinal functions; use of over-the-counter medications for GI symptoms in the seven-day period prior to the study. The procedures, purposes, and risks of participation in the study were explained, and informed written consent was obtained from the subjects involved. The study was approved by the Ethics Committee of the "Federico II" University of Naples. The study was also supported by The Beverage Institute for Health & Wellness, The Coca Cola Company, L.L.C., Atlanta, USA.

**Table 1:** Demographic characteristics of subjects at recruitment

Subject	Gender	Age	BMI
A	M	24	22,6
B	F	23	22,3
C	F	20	22,8
D	F	19	24,1
E	F	24	20,4
F	M	22	24,8
G	M	23	23,5
H	M	24	23,9
I	F	21	24,1
J	M	21	21,4

BMI: Body Mass Index.

### General Design

**Figure 1:**
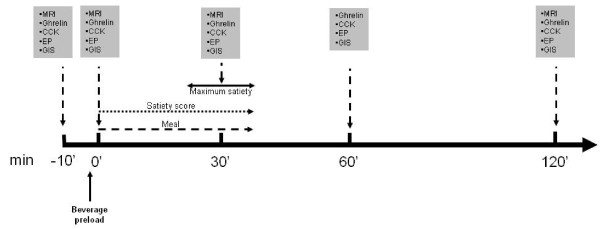
**General design of the experiment**. Gastric magnetic resonance (MRI) was performed at basal time (-10 min), after a beverage pre-load, at maximum satiety and at 120' minutes after the start of the meal. A blood sample was taken to assay ghrelin and cholecystokinin (CCK), measure gastrointestinal symptoms (GIS) (postprandial fullness, early satiety, nausea, bloating, epigastric pain, epigastric burning) and eating perceptions (EP) (hunger, desire to eat, prospective of food consumption); this was performed at the same times and also 60 mins following the start of the meal.

The subjects performed the experiment six times, three times with one standardized solid meal and three with a liquid one. Each experiment was performed at almost one week interval. Both solid and liquid meals were administered following a pre-administration of 300 ml of still water, a commercial non-caloric de-carbonated or a carbonated beverage (Sprite Zero^®^). Apart from carbonated water, the ingredients of the commercial beverage included sweeteners (aspartame 40 mg/100 ml; acesulfame K 40 mg/100 ml), flavors (lemon and lime aromas 100 mg/100 ml) and acidity regulators (citric acid 230 mg/100 ml; trisodium citrate 10 mg/100 ml). The carbon dioxide concentration in the carbonated beverage was around 3.7 volume when the bottle was opened, equaling to 1125 ml of CO_2 _in the beverage consumed. The sequences of experiments (solid or liquid meal; beverage type) were random but the order of the experiments was balanced to avoid that any one sequence prevailed over another. All the beverages used in the study were colorless and were contained in similar transparent 300 ml bottles, and numbered with a key that was decoded only at the end of each study. The bottles were stored at 4° C and the beverages administered at 10-12°C directly from the bottle. The subjects were studied in the morning following an overnight fast lasting at least 10 hours. These subjects answered standardized questionnaires regarding their eating perceptions and satiety score. They drunk the 300 ml beverage in 3 minutes, consumed the meal at an established rate (see below) until maximum satiety was reached, and then performed a gastric magnetic resonance and a hormonal assay at defined intervals (see Figure 1). Upon recruitment, the subjects were screened for upper GI symptoms using standardized questionnaires. All subjects underwent satiety tests with liquid and solid meals immediately after the pre-administration of 300 ml of still water (water), a non-caloric de-gassed beverage (B-CO_2_) and a non-caloric carbonated beverage (B+CO_2_). The B-CO_2 _beverage was de-gassed by one of the authors (CC) immediately prior to the experiment, by means of an ultrasound procedure (Elmasonic S - Ultrasonic Unit, Singen, Germany), and the experiment was performed by two other authors (MFS and LV) unaware of the content of the beverage.

### Symptoms Questionnaire

All subjects evaluated gastrointestinal symptoms and eating perceptions prior to beverage consumption, immediately before beginning the meal (0') and 30, 60 and 120 minutes after beginning the meal (see Figure 1). The symptoms evaluated were postprandial fullness, satiety, nausea, bloating, epigastric pain and epigastric burning; whereas eating perceptions were hunger, the desire to eat and the prospective of food consumption. Measurements were performed by means of a Visual analogue scale (VAS) calibrated to100 mm.

### Satiety Liquid Meal Test

According to the standard procedure, a peristaltic pump (Minipuls2; Gilson, Villiers-Le-Bel, France) filled one of two beakers with a liquid meal (Nutridrink, Nutricia; 49% carbohydrate, 35% fat, 16% protein, caloric density 1.5 kcal mL) at a rate of 15 ml/min. The subjects were asked to maintain the intake at the filling rate (112 Kcal/5 minutes), thereby alternating the beakers as they were filled and emptied. During the five-minute intervals the subjects were free to drink. At the end of each five minute period, they scored their satiety using a graphic rating scale that combines verbal descriptors on a scale from 0 to 5 (1 = threshold, 5 = maximum satiety). The participants were instructed to stop meal intake when a score of 5 was reached [17,18].

### Satiety Solid Meal Test

The standard meal included various food items, i.e., white bread, cheese, ham spread (Spuntì, Kraft Foods, Italy). The composition of this meal was almost similar to that of the liquid meal (50% carbohydrate, 31% fat, 19% protein). The subjects were asked to ingest a constant number of Kcals at 5 min. intervals (110 kcal/5 min) administered as standardized portions of sandwich, and during these intervals they were free to eat each portion at the rate they chose. The subjects scored their satiety levels on a visual analogue scale that combined verbal descriptors rated from 0 to 5 (1 = threshold, 5 = maximum satiety). The participants were instructed to stop eating when a score of 5 was reached.

### Magnetic Resonance Imaging (MRI) for gastric Volume Study

All subjects underwent an anatomical three-dimensional acquisition on a 1.5 T MRI system (Philips Medical Systems, Intera). During the MRI, each subject was positioned lying on his/her back at a 15° angle. As for parallel imaging, method sensitivity encoding was applied to increase image acquisition rate. Four acquisitions were performed for each subject: at baseline (t_0_), after drinking the beverage (t_1 _= 0), at maximum satiety (t_2_) and at the end of the experiment (t3 = 120 min), for both the liquid and the solid meals and for each of the three different beverage types (water, B-CO_2 _and B+CO_2_), for a total of 24 three-dimensional acquisitions for each subject. Gastric volumes were determined by means of MR images acquired on a transverse plane (up to 50 contiguous transverse slices, 5 mm thickness, resolution 1.3021 × 1.3021 mm, echo time 1.95 ms, repetition time 3.9 ms, no gap, acquisition matrix 224 × 256, flip angle 60°). An abdominal send-receive coil was wrapped around the abdomen for signal detection.

After automatic segmentation [19], surface reconstruction and three-dimensional stomach contours rendering, gastric meal volume and gastric gas volume areas were computed using the sum of voxels across all slices. Stomach volume was calculated by summing the pixels outlined in each bi-dimensional image slice and by integrating the sum of all slices [20,21]. In each image slice, intragastric gas could be identified by the distinct null signal intensity compared to the meal contents. The sum of the pixels reflecting intragastric gas contents, integrated by the sum of all slices, yielded the subject's gas volume. Meal volume was determined by subtracting intragastric gas volume from the stomach volume.

A three-dimensional representation of the stomach based on the contours outlined was used to separate the stomach volume into proximal and distal gastric volumes. The stomach was divided into proximal and distal parts by identifying the incisura angularis on the lesser curvature and by drawing a line across the incisura angularis perpendicular to the great curvature of the stomach [21]. In particular, the proximal and distal gastric regions were identified by three dimensional reconstructions of the stomach, divided at the angulus. Stomach volume (total, proximal, distal and intragastric gas expressed in millilitres) was compared in all conditions and at all time-points.

### Biochemical Analysis

Plasma samples were obtained in centrifuge tubes containing aprotinin and were stored at -80°C immediately after centrifugation at 4°C until analysis. Glucose levels were measured using routine methods. Plasma total immunoreactive ghrelin and CCK ([26-33] non-sulfated form) were measured by enzyme immunoassay. Ghrelin was measured in duplicate using commercial ELISA kits (Phoenix Pharmaceuticals, Belmont, CA); the inter- and intra-assay coefficients of variance were < 10%. The lower and upper detection limits for this assay were 0.12 ng/mL and 100 ng/mL. CCK ([26-33] octapeptide non-sulfated form) was measured in duplicate using a commercial ELISA kit (Phoenix Pharmaceuticals, Belmont, CA); the inter- and intra-assay coefficients of variance were < 10%, with a lower detection limit of 0.04 ng/mL [22,23].

### Data Analysis

A preliminary evaluation of satiety data variability was performed for sample size calculation. Based on our previous data [*18*] relating to a satiety drinking test, we calculated the sample size and relative statistical power of this study. Assuming that alpha is 0.05, the number of groups 3 and the effect size f 1.25, we extrapolated the following statistical power (1-β error probability) for each sample size: 0.75 for 8 cases; 0.85 for 9; 0.92 for 10; 0.95 for 11.

Total gastric volume was evaluated by calculating the sum of the voxels in all the slices studied by MRI and the data was expressed in ml. Gastric volumes are calculated both as absolute value and, to correct individual variation, also as difference (delta value) with respects to the basal value (prior to beverage consumption).

Ghrelin and CCK kinetics were evaluated taking into consideration the values obtained as difference with respects to the basal value (prior to beverage consumption). The area under the curve was also evaluated for both hormones, calculating curve interpolating times at 0, 30, 60 and 120 min. The ghrelin nadir and CCK peaks were respectively calculated from the kinetic curves.

Many statistical calculations were carried out using paired repeated-measures analyses of variance (ANOVA) as well as Tukey's multiple comparison post-test. To examine the difference between solid and liquid meals, we performed a paired T-test for each experiment carried out with each beverage. The results are reported as mean ± SEM.

## Results

### Symptoms during meal intake

The subjects did not suffer from any relevant pathological symptoms such as postprandial fullness, nausea, bloating, epigastric pain or epigastric burning during the experiment with either the solid or the liquid meal, or following any of the beverages. A non significant increase in satiety was found only immediately after carbonated beverage (Water: 32 ± 6 mm; B-CO_2_: 31 ± 4; B+CO_2_: 40 ± 6). Moreover the desire to eat (Water: 59 ± 7 mm; B-CO_2_: 61 ± 7; B+CO_2_: 52 ± 6), hunger (59 ± 7; 60 ± 7; 52 ± 6) and the prospective of food consumption (61 ± 7; 62 ± 7; 56 ± 6), were transiently but non significantly decreased immediately (T_0_) after the carbonated beverages. No differences were found between the meals during the other periods of the experiments with any of the three beverages.

### Satiety test

**Figure 2:**
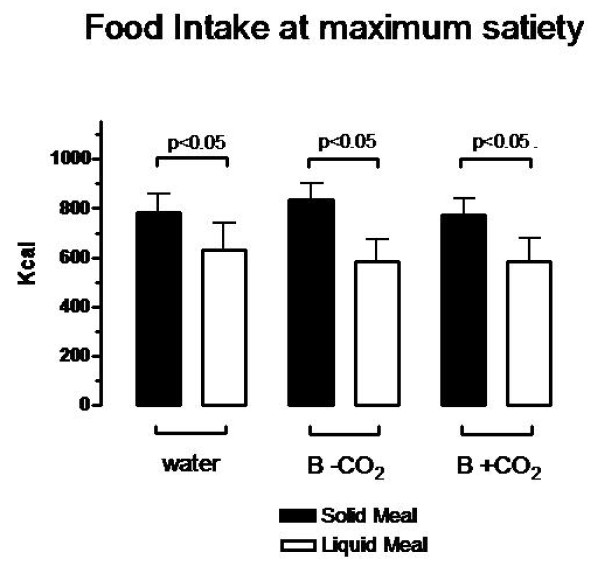
**Meal intake at maximum satiety is expressed in kcal**. The test was performed after a beverage pre-load administering a solid or liquid meal at a constant rate (about 110 kcal) every five minutes until maximum satiety. No differences were found between water, a de-carbonated beverage (B-CO2) and a carbonated beverage (B+CO2) for both solid and liquid meals. Significant differences were found in each beverage in both solid and liquid meals.

Meal intakes at maximum satiety did not differ between the three experiments performed with the different beverages, either with the solid (Water: 783 ± 77 kcal; B-CO_2_: 837 ± 66; B+CO_2_: 774 ± 66) or the liquid meal (630 ± 111; 585 ± 88; 588 ± 95) respectively (see Figure 2). The difference analysis between the meals showed a significant increase (p < 0.05) in solid meal compared to liquid meal intake in all comparisons with each beverage.

### Gastric Volume

**Figure 3:**
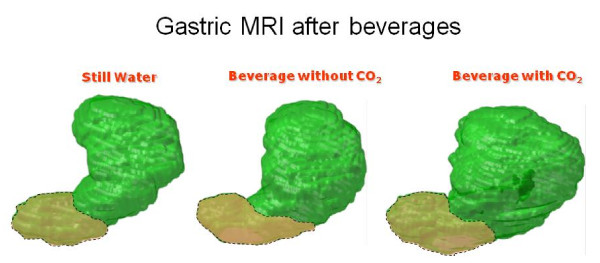
**Gastric shape evaluated by MRI in one subject immediately after beverage intake**. The shape, particularly of the proximal stomach (green), appears enlarged after the consumption of a beverage containing carbon dioxide.

**Figure 4:**
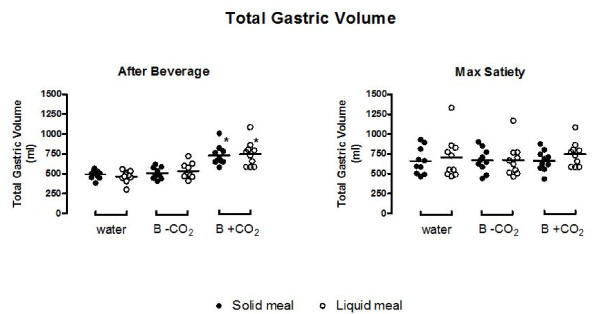
**Total gastric volume evaluated with MRI after beverages and at maximum satiety**. Volumes are significantly increased only immediately after the consumption of a beverage containing carbon dioxide in both groups subsequently administered solid and liquid meals. At maximum satiety, the volumes are similar in all groups. B-CO2: beverage without carbon dioxide; B+CO2: beverage with carbon dioxide. * p < 0.05 vs water and B-CO2.

Absolute total gastric volume (TGV) significantly (p < 0.05) increased immediately after a 300 ml intake of B+CO_2 _with respects to the intake of water and B-CO_2 _(see Figures 3 and 4). However, we found no difference in TGV between experiments at maximum satiety with any of the three beverages, with either the solid or the liquid meal.

The corrected value of gastric volume expressed as difference from basal value (TGVd) also showed a similar trend following carbonated beverage consumption. Indeed, the increases in TGVd after B+CO_2 _with respects to water and B-CO_2 _were about 250 ml and were mainly characterized by the gas contained in the B+CO_2 _(see Table 2). The analysis of the corrected (difference from basal value) proximal and distal volume confirmed TGVd data, showing a significant increase in both proximal and distal volume following the consumption of B+CO_2 _compared to water and B-CO_2 _(see Table 2).

**Table 2:** Total Gastric Volume (TGVd), Gas Gastric Volume (GGVd), Proximal Volume (PVd) and Distal Volume (DVd) immediately after the consumption of a 300 ml beverage during the experiments carried out with solid and liquid meals

	*Solid Meal Experiment* *(ml)*				*Liquid Meal Experiment* *(ml)*			
	**TGVd**	**GGVd**	**PVd**	**DVd**	**TGVd**	**GGVd**	**PVd**	**DVd**

**Water **[10]	282 ± 13	64 ± 8	249 ± 16	33 ± 7	267 ± 17	42 ± 8	237 ± 18	30 ± 5
**B - CO**_**2 **_[10]	302 ± 17	63 ± 11	277 ± 15	25 ± 6	354 ± 19	69 ± 12	320 ± 17	32 ± 5
**B + CO**_**2 **_[10]	548 ± 30*	268 ± 29*	483 ± 27*	66 ± 6*	558 ± 41*	229 ± 39*	503 ± 37*	58 ± 8*

The values are expressed as differences from basal volumes (Delta Volume) and as mean ± SE.

B - CO_2_: Beverage without carbon dioxide; B + CO_2_: Beverage with carbon dioxide; the number of experiments is indicated in brackets; *p < 0.05 vs water and B - CO_2._

TGVd at maximum satiety (see Table 3) and at 120 min (Solid meal: Water 347 ± 51 ml; B-CO_2 _369 ± 34; B+CO_2 _335 ± 27; Liquid meal: 158 ± 44; 145 ± 40; 157 ± 47) did not differ between the experiments with the three beverages within the context of the type of meal. However, a significant difference (p < 0.05) was found at 120 minutes between the two types of meal after all pre-meal beverages with a lower TGVd during the liquid meal respect to the solid one. Moreover, in all the experiments with the solid and liquid meals and the beverages, a similar proximal value was found upon maximum satiety (see Table 3). On the other hand, distal volume was significantly (p < 0.05) increased during the solid meal compared to the liquid one, but no differences were found between beverages for each meal (solid or liquid) experiment.

**Table 3:** Total (TGVd), Proximal (PVd) and Distal Gastric (DVd) Volumes at maximum satiety following a 300 ml beverage during the experiments carried out with solid and liquid meals

	*Solid Meal Experiment* *(ml)*			*Liquid Meal Experiment* *(ml)*		
	**TGVd**	**PVd**	**DVd**	**TGVd**	**PVd**	**DVd**

**Water **[10]	456 ± 54	395 ± 45	65 ± 18	510 ± 85	467 ± 80	34 ± 6*
**B - CO**_**2 **_[10]	466 ± 40	407 ± 33	59 ± 14	493 ± 63	464 ± 58	24 ± 6*
**B + CO**_**2 **_[10]	484 ± 35	420 ± 29	63 ± 9	513 ± 78	477 ± 70	28 ± 5*

Values are expressed as differences from basal volumes (Delta Volume) and as mean ± SE.

B - CO_2_: Beverage without carbon dioxide; B + CO_2_: Beverage with carbon dioxide; the number of experiments is given in brackets; *p < 0.05 vs Distal Volume of Solid Meal Experiment.

### Ghrelin, cholecistokinin and glucose

There was no difference in glucose kinetics between all the experiments with the three beverages and the two types of meals (data not shown).

**Figure 5:**
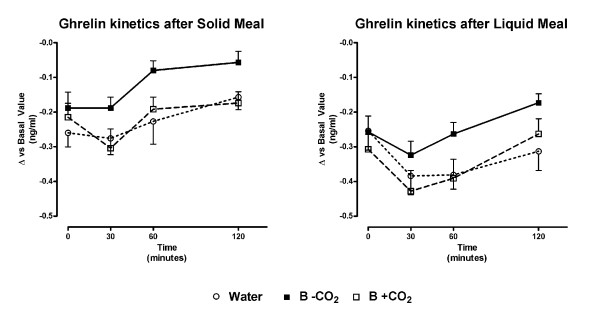
**Ghrelin kinetics after solid (left) and liquid (right) meals**. Data is expressed as difference vs basal level before beverage and meal (mean ± SE). Time 0 was considered as time after beverage intake. B-CO_2_: beverage without carbon dioxide; B+CO_2_: beverage with carbon dioxide. No significant differences were found by means of the ANOVA analysis between kinetic values between beverages. However, the area under curve analysis (see Table 4) showed a lower ghrelin decrease in B-CO_2 _with respects to water and B+CO_2_, which proved to be significant (p < 0.05) during the solid meal.

The analysis of the ghrelin curve showed a similar decrease in the values of this hormone following the consumption of each beverage and meal (see Figure 5). The de-gassed beverage induced a significantly lower ghrelin decrease in the area under curve (AUC) only during solid meals (see Table 4). The comparison between solid and liquid meals showed a significantly lower AUC with the solid meal than with the liquid one after all three beverages (see Table 4).

**Table 4:** Area under curve (AUC) of ghrelin and cholecistokinin (CCK) following a 300 ml beverage during the experiments carried out with solid and liquid meals

	*Ghrelin AUC*			*CCK AUC*		
	**Water**	***B - CO2***	***B + CO2***	**Water**	***B - CO2***	***B + CO2***

**Solid meal **[10]	-27.1 ± 5.1^A,a^	-13.8 ± 3.3 ^B,a^	-26.2 ± 4.5 ^A,a^	58.5 ± 11.3^A,a^	66.3 ± 19.1^A,a^	98.9 ± 13.7^A,a^
**Liquid meal **[10]	-41.9 ± 5.2^A,b^	-30.6 ± 3.6 ^A,b^	-42.9 ± 5.8 ^A,b^	85.5 ± 13.8 ^A,a^	82.9 ± 18.7 ^A,a^	62.6 ± 9.2 ^A,a^

Values are expressed as ng/ml/min and as mean ± SE.

Capital and small letters express differences between beverages and meals respectively. Values not sharing a common superscript letter are significantly different (p < 0.05). The number of experiments is given in brackets.

**Figure 6:**
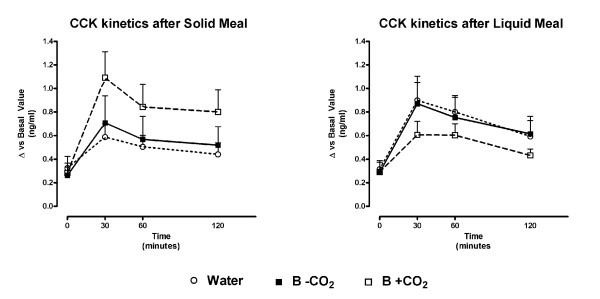
**Cholecystokinin (CCK) kinetics after solid (left) and liquid (right) meals**. Data is expressed as difference vs basal level before beverage and meal (mean ± SE). Time 0 was considered the time after beverage intake. B-CO_2_: beverage without carbon dioxide; B+CO_2_: beverage with carbon dioxide. No significant differences were found by means of the ANOVA analysis between kinetic values between beverages.

Kinetic CCK curves showed no significant difference between beverages (see Figure 6). However, the carbonated beverage determined a trend to a higher but non significant AUC level during the solid meal compared to the other beverages (see Table 4). On the contrary, the carbonated beverage had a non-significant trend towards a lower AUC during the liquid meal compared to the effect of water and B-CO_2 _(see Table 4). The comparison between meals showed a non significant trend toward an increase in the CCK AUC after B+CO_2 _during the solid meal compared to the same experiment carried out with the liquid meal.

## Discussion

This study was performed on healthy non-obese subjects to verify the effect of a 300 ml non-caloric, commercial carbonated or de-gassed beverage, on satiety compared to water. The main methodological interest of this article is the contemporary evaluation of satiety, gastric volume by means of a non-invasive method, and some gastrointestinal hormones involved in food intake control. Mainly, we found a clear increase in gastric volume immediately after the consumption of a carbonated beverage without any influence whatsoever on food intake. Therefore, in a normal subject, carbon dioxide at its maximum concentration (3.7 volume) contained in a 300 ml beverage consumed 3 min prior to the meal did not seems to influence satiety or food intake compared to the consumption of still water or the same beverage without carbon dioxide. This study also showed that a carbonated beverage does not modify the quantity of solid or liquid food consumed. In short, the intake of a solid meal remains unaltered if the subject, prior to consuming such food, drinks the same quantity of water, carbonated or de-gassed beverage. The same occurs with a liquid meal. However, some slight differences were found in hormone kinetics, likely related to both meal consistency and beverage carbonation.

The increased gastric volume following carbonated beverage consumption found in our study was a predictable result, yet the lack of any influence on food intake following the consumption of a carbonated beverage is intriguing. The process that limits meal size derives from a coordinated series of neural and humoral signals that originate from the gut in response to the mechanical and chemical properties of the food ingested [24]. Among these factors, a mechanical distension has been described as a relevant aspect [25,26]. In our study, the increase in gastric volume following the consumption of a carbonated beverage appeared to be related to the gas content of the beverage. However, we observed a similar increase in gastric volume after carbonated beverage consumption and at maximum satiety with respects to the basal value (~500 ml). These data suggest a different hypothesis to explain the lack of satiety following the consumption of the carbonated beverage. Firstly, the duration of gastric distension due to carbon dioxide can be very short due to either the absorption of gas by the gastric wall or its rapid elimination by eructation. Moreover, the absence of nutrients in the non-caloric beverage could explain the lack of other factors (i.e. hormone release) involved in satiety [24].

The level of beverage carbonation was shown to affect food intake only with consumptions exceeding 300 ml. Moorhead et al compared the effects of identical sugar-sweetened beverages (400 ml; 639 kJ), with three levels of carbonation, consumed 10 min before an ad libitum lunch. These results showed that the beverages with higher carbonation led to higher satiety until lunch and lower energy intakes at lunch [9]. These authors concluded that the level of carbonation affects satiety and subsequent intakes in the short term, and speculated that this may be due to effects on gastric distension. In our study we used a 300 ml non-caloric pre-meal beverage with 3.7 volume of carbon dioxide and did not find any interference with the intake of a meal consumed at a constant rate.

We also found earlier satiety during consumption of a liquid meal compared to a solid meal, regardless of the beverage administered. The two meals (solid and liquid) were isocaloric but the liquid meal had a slightly increased fat content while the solid one had a higher protein content usually considered to be more satiating than fat [27,28]. Therefore it is likely that the satiating effect observed may be mainly due to meal consistency or meal volume rather to its nutrient content. Our results showed that gastric volumes at maximum satiety were similar for both the solid and the liquid meals. Therefore, the consistency of the meal and particularly its viscosity can be a relevant cause that triggered satiety [29].

The gastric regional meal distribution showed that the relevant effect on gastric distension seemed to affect the proximal gastric region. However, distal volume at maximum satiety decreased during the liquid meal compared to the solid one. This finding can explain some differences in food intake between solid and liquid meals, and supports the hypothesis of a different physical gastric distribution or an increased gastric emptying rate of a liquid compared to a solid meal, without any influence related to beverage type.

The analysis of hormone kinetics showed a decrease in ghrelin expressed as area under the curve during the liquid meal compared to the solid one after all beverage consumption. Our results support the hypothesis that, apart from a more rapid gastric emptying, also an increased interaction of nutrients with the taste receptors of ghrelin cells is able to limit liquid food intake [15]. Moreover, by analyzing the effect of the beverages, we observed that water and non-caloric carbonated beverage pre-load administered before a solid meal determined a significant decrease in ghrelin compared to a de-gassed beverage. Therefore, a sweetened non-caloric and de-gassed beverage determined a significantly lower meal-induced ghrelin decrease compared to water. The carbonation of the non-caloric beverage was able to amplify ghrelin decrease until the level determined by the water pre-load was reached. A similar, yet non significant effect was observed during the consumption of the liquid meal.

Ghrelin levels increase with fasting and decrease after meals [30]. In addition to fasting, ghrelin expression can be stimulated in rats by means of insulin-induced hypoglycemia [31], and some observations indicate a direct inhibitory effect of glucose on ghrelin-containing cells in the oxyntic mucosa [32]. On the basis of these concepts and considering that, in our experiments, glucose levels (data not shown) were similar in all beverages and meals, we can speculate that the sweeteners (aspartame and acesulfame K) were able to decrease the effect of nutrients on the cells containing ghrelin, by means of a receptor-competition mechanism [33-35]. Carbonated beverages seem to determine a more significant negative feedback relating to ghrelin secretion. This effect appeared more evident during the solid meal compared to the liquid one. Also, we can speculate that the presence of carbon dioxide in the beverage, with a consequently increased acidity [15,36], was able to improve the interaction between glucose or sweeteners and ghrelin cell. Moreover, the sour sensing taste receptors present in the stomach could possibly mediate the effect on ghrelin release. Both carbonated drinks and acids can interact via carbonic anhydrase with a member of the transient receptor potential (TRP) ion channel family PKD2L1 (poly kidney disease-2-like 1) [15,36]. However, despite the difference found in our results, no effect on amount of food intake was observed. Perhaps, other conditions, such as increased beverage volume or meal quality, could modify food intake by means of carbonated and de-gassed non-caloric beverages, by amplifying the effect on ghrelin secretion.

## Conclusion

Our study showed, in healthy subjects, a clear increase in gastric volume following a 300 ml pre-meal carbonated beverage without any influence whatsoever on food intake whether the consistency of the meal was solid or liquid. A liquid consistency induces an earlier satiety and a decreased food intake with respects to a solid consistency, regardless of the pre-meal beverages. Interestingly, the consistency of the meals and the carbonated beverages seemed to influence ghrelin releases, which was unable, under our experimental conditions, to modify the amount of food intake in healthy subjects. On the other hand, hormone release and satiation could be significantly modified by different administration times, and food and carbonated beverage size combinations influencing food intake. Further studies are needed to analyze if other food and beverage combinations are able to modify satiation.

## List of abbreviations

CO_2_: Carbon dioxide; B+CO_2_: Carbonated beverage; B-CO_2_: De-Carbonated beverage; SM: Solid meal; LM: Liquid meal; CCK; Cholecystokinin; BMI: Body mass index; TGV: Total gastric volume; TGVd: Delta total gastric volume (difference from basal value); MS: Maximum satiety; ANOVA: Analysis of variance; SEM: Standard error of the mean; AUC: Area under curve; VAS: Visual analog scale; MRI: Magnetic resonance imaging; ELISA: Enzyme linked immunosorbent assay.

## Competing interests

RC has received research funds from The Coca-Cola Company, Atlanta, USA. MB is employer of The Coca-Cola Company, Atlanta, USA. All other authors declare that they have no competing interests.

## Authors' contributions

RC, MFS and GS were responsible for the study concept and design.; RC drafted the manuscript; RC, MFS, GS, FPZ, EE and AA contributed to the acquisition and analysis of the data; EN and AA performed gastric MRI; CC and LV performed hormone analysis; MB assisted with data analysis and interpretation of findings; MFS, FPZ, VV and MB and provided critical revision of the manuscript. All authors read and approved the final manuscript.

## Authors' Information

RC is Associate Professor of Gastroenterology; MFS is Gastroenterologist and PhD; GS is Assistant Professor of Gastroenterology and PhD; LV is Medical Doctor and Fellow; CC is Pharmacist, PhD and Post-doc fellow; FPZ is Medical Student; VV is Nutritionist; EE is Nurse; EN is Radiologist; AA is Electronic Engineer; MB is Medical Doctor and Director of Health and Wellness Programs Global Scientific and Regulatory Affairs of The Coca-Cola Company, Atlanta, USA.
